# Supplementary material for: Application Route and Immune Status of the Host Determine Safety and Oncolytic Activity of Oncolytic Coxsackievirus B3 Variant PD-H
Source: Viruses. 2021 Sep 24;13(10):1918. doi: 10.3390/v13101918 (PMC8539752; doi:10.3390/v13101918)
Supplement: Supplementary file 1 [file viruses-13-01918-s001.zip › viruses-1363453-supplementary.pdf]

viruses

MDPI

Supplementary Data

Gene	Position	Mutations compare to PD (Genbank accession no: AF231765)
**5' UTR**	1 - 742 nt	610. nt C → T
**VP4**	743 - 949 nt	881. nt C → T (VP4 47. aa T→A)
**VP2**	950 - 1738 nt	No differences
**VP3**	1739 - 2452 nt	1801. nt A→G (VP3 34. aa T→M)
		1839. nt C→T (silent mutation)
		2448. nt C→A (VP3 237. aa F→Y)
**VP1**	2453 - 3295 nt	2965. nt A→G (silent mutation)
**2A**	3296 - 3745 nt	No differences
**2B**	3746 – 4042 nt	No differences
**2C**	4043 – 5029 nt	4078. nt A→G (silent mutation)
		4165. nt A→G (silent mutation)
		4167. nt G→A (2C 42. aa G→E)
		4878. nt A→G (silent mutation)
**3A**	5030 – 5296 nt	No differences
**3B**	5297 – 5362 nt	No differences
**3C**	5363 – 5911 nt	No differences
**3D**	5912 – 7300 nt	7169. nt C→T (silent mutation)
		7174. nt A→G (silent mutation)
**3' UTR**	7301 – 7400 nt	No differences

Table S1. Nucleotide and amino acid differences between CVB3 variant PD (Gene bank accession number: AF231765) and the cDNA clone of PD-0 (PD-H). PD-H contains following nucleotide differences relative to PD: C610T, C881T, A1801G, C1839T, C2448A, A2965G, A4078G, A4165G, G4167A, A4878G, C7169T and A7174G. (First letter indicates the nucleotide according to GenBank sequence, last letter indicates the nucleotide according to our sequence, number indicates the position of nucleotide). nt: nucleotide. aa: amino acid. UTR: untranslated region. Note: One nucleotide difference (C881T) was identified between PD-H and PD-0.

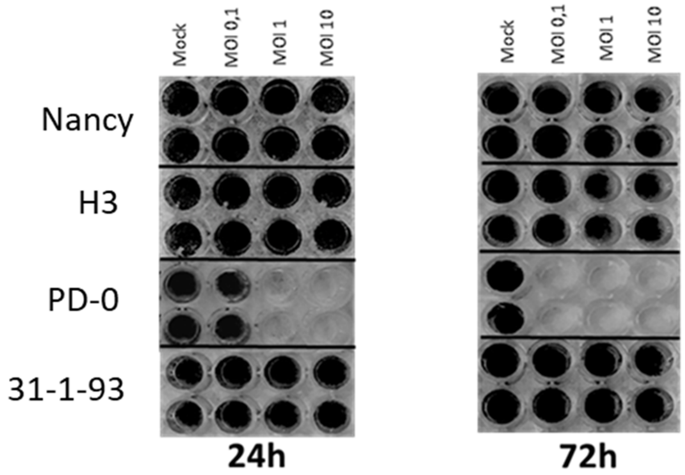

Supplementary Figure S1

Cytotoxicity of CVB3 strains Nancy, H3, PD-0 and 31-1-93 in the colorectal carcinoma cell line Colon-26. Colon-26 cells grown in 96-well plates were infected with indicated virus strains and MOIs and analyzed for inducing of cytotoxicity 24 h and 72 h later by staining the cells with crystal violet.

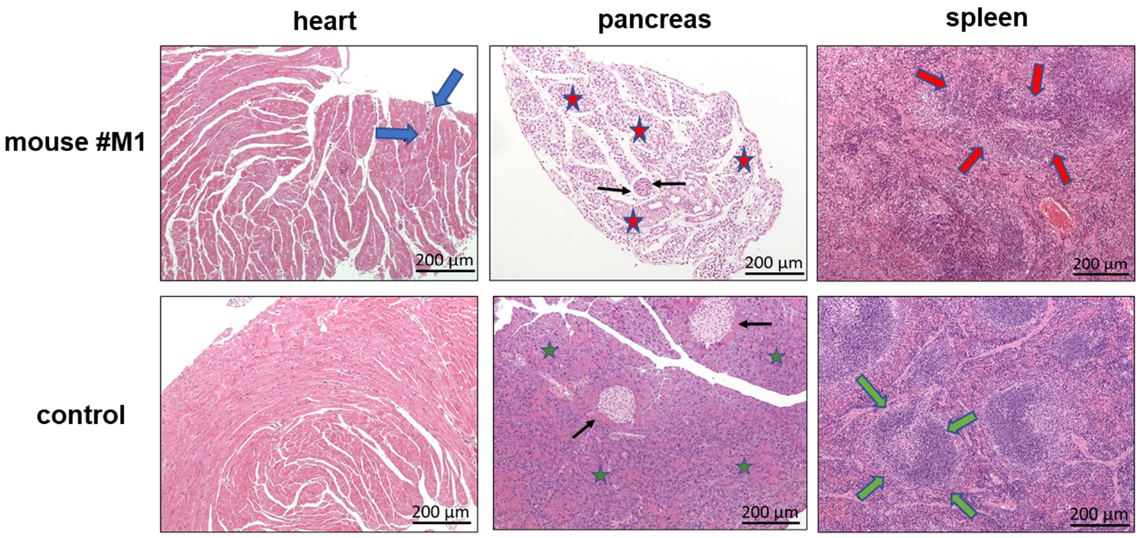

Supplementary Figure S2

Side effects induced by PD-H after intratumoral injection into DLD1 tumors of mouse #M1. Balb/C mice were injected with 5x10^5 DLD-1 cells into both flanks. When the tumor sizes reached ~0.5 cm diameter, one of the tumors was injected with 3x10^6 pfu PD-H. Mouse #1 was sacrificed 13 days after tumor cell administration because of poor health. Tissue slides were stained with H&E. Blue arrows, reveal small, focal areas of inflammation and myocyte damage in the heart; black arrows, no damage of islets of Langerhans in the pancreas; red stars, damaged exocrine pancreas tissue; green stars, intact pancreas tissue; red arrows, rarefication of spleen follicles; green arrows normal follicles of spleen.

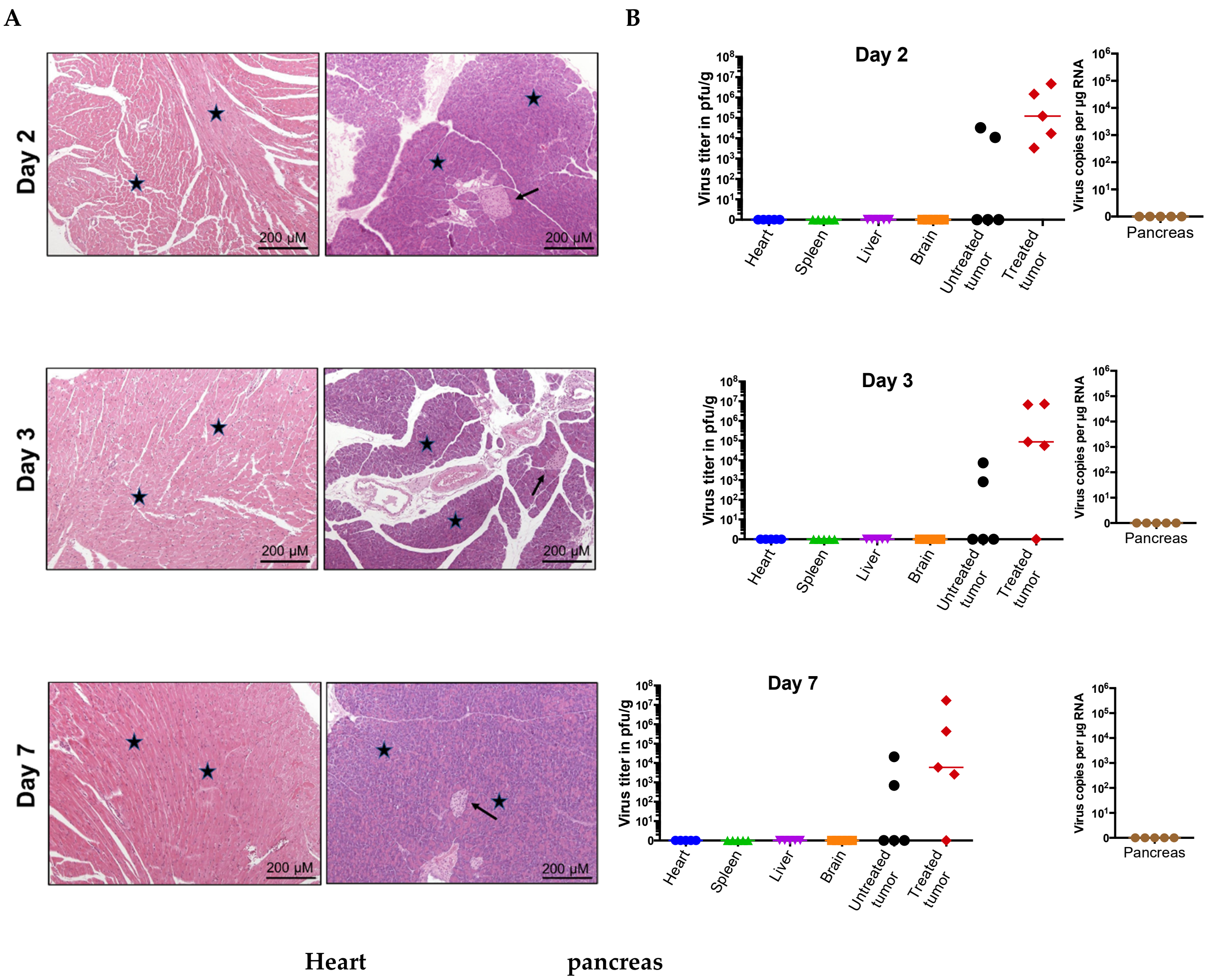

Supplementary Figure S3

Biodistribution of PD-H after intratumoral injection into subcutaneous Colon-26 tumors of Balb/C mice. Balb/C mice were injected with 2x10^5 Colon 26 cells into both flanks. When the tumor sizes reached ~0.5 cm diameter, one of the tumors was injected with 1x10^6 pfu PD-H (n=5). Animals were sacrificed 2, 3 and 7 days after virus injection. (**A**) The pancreases and the hearts were histologically investigated after staining the tissue with H&E. Black stars, normal tissue; black arrow, islet of Langerhans. (**B**) Virus load was determined by plaque assay in the organs and in the injected and non-injected contralateral tumors (*left diagrams*). In the pancreas, the virus RNA copy number was determined by real time RT-PCR (*right diagrams*). The data are shown for each animal and as medians for each group.

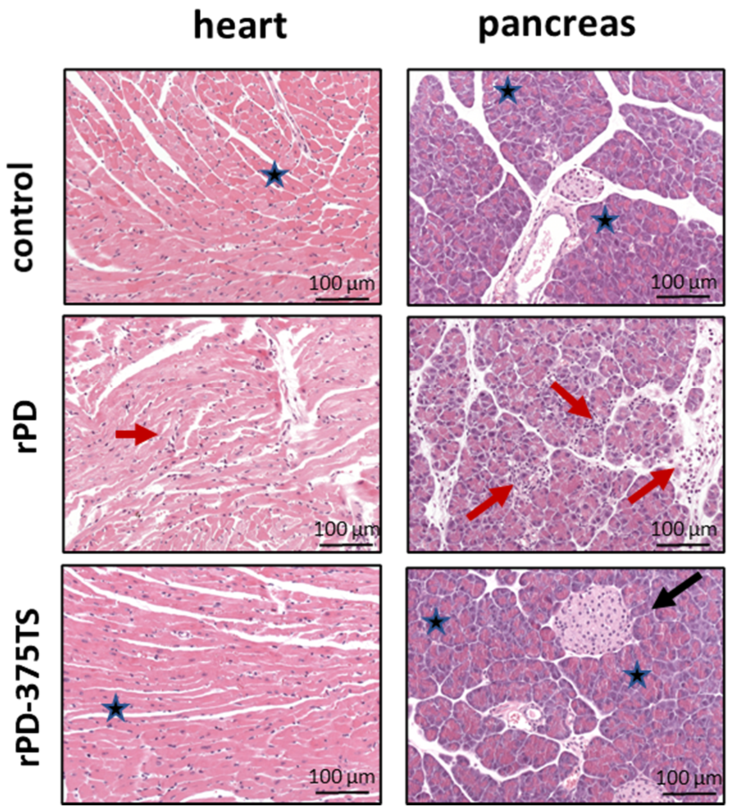

Supplementary Figure S4

Balb/C mice infected intraperitoneally (i.p.) with PD-H-375TS do not develop pancreatitis and myocarditis. Balb/C were i.p. infected with each $5x10^6$ pfu PD-H (n=4), PD-H-375TS (n=4) or injected with PBS (n=2) at three consecutive days. Animals were sacrificed 7 days after the first virus injection and organs were histologically examined after staining with H&E. black stars, normal tissue; black arrow, islet of Langerhans; red arrows, tissue areas with mononuclear cell infiltration.
